# Supplementary material for: Ultraviolet light-induced collagen degradation inhibits melanoma invasion
Source: Nat Commun. 2021 May 12;12:2742. doi: 10.1038/s41467-021-22953-z (PMC8115293; doi:10.1038/s41467-021-22953-z)
Supplement: Supplementary file 1 — Supplementary Information [file 41467_2021_22953_MOESM1_ESM.pdf]

Supplementary Information

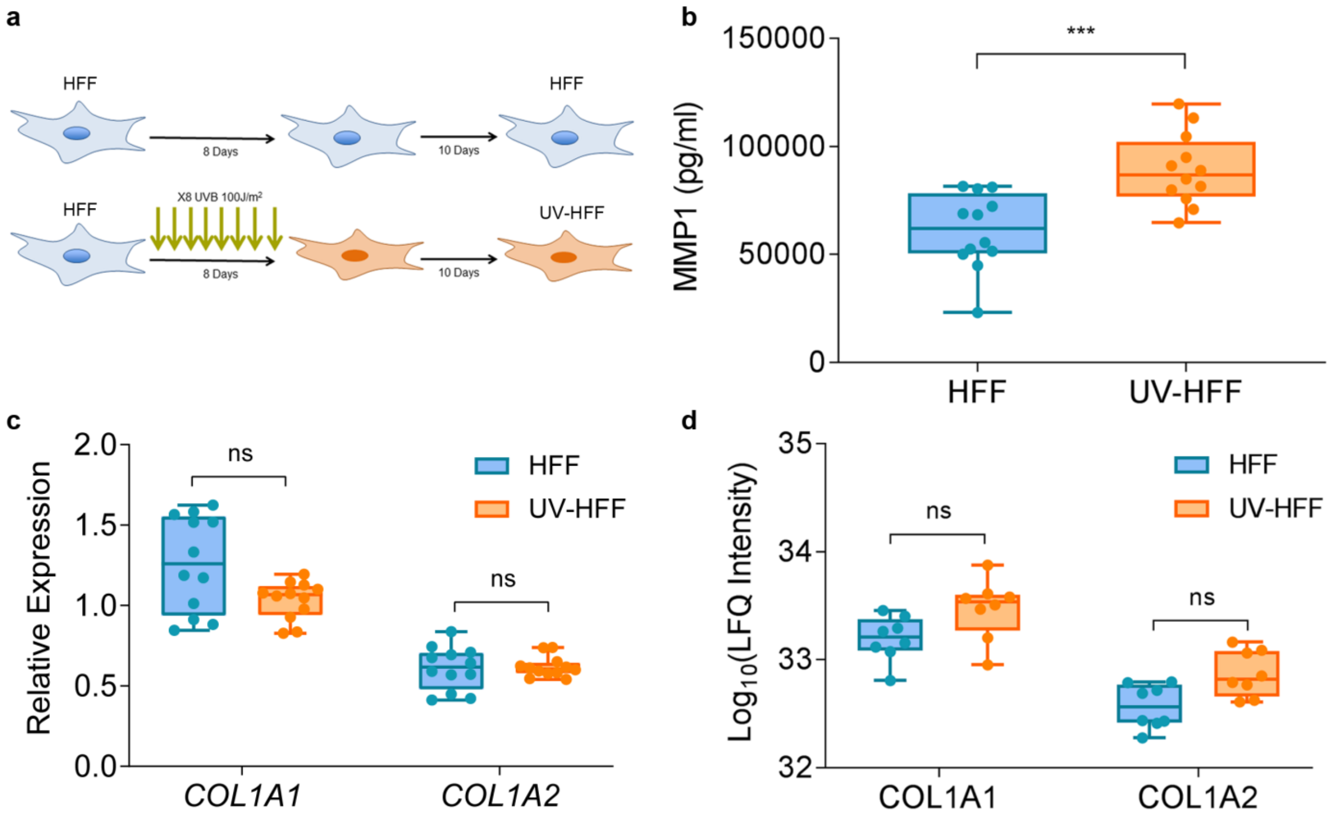

Supplementary Figure 1. UVR damaged fibroblasts upregulate MMP1 but not COL1A1. a. Graphic representation of the generation of isogenic HFF and UV-HFF by chronic UVR treatment. **b.** Quantification of MMP1 in the secretome of HFF and UV-HFF fibroblasts, (two-sided Mann Whitney U ***p=0.0009). **c.** Relative Expression of *COL1A1* and *COL1A2* in HFF and UV-HFF fibroblasts, (two-sided Mann Whitney U ns: not significant). **d.** Label free quantification (LFQ) of COL1A1 AND COL1A2 in the HFF and UV-HFF matrisome by mass spectrometry, (two-sided Mann Whitney U ns: not significant). All data represents duplicate samples collected from biological replicate cell lines, box plot showing biological and technical replicate points. box plots represent 25th to 75th percentiles with median, whiskers represent minimum and maximum values.

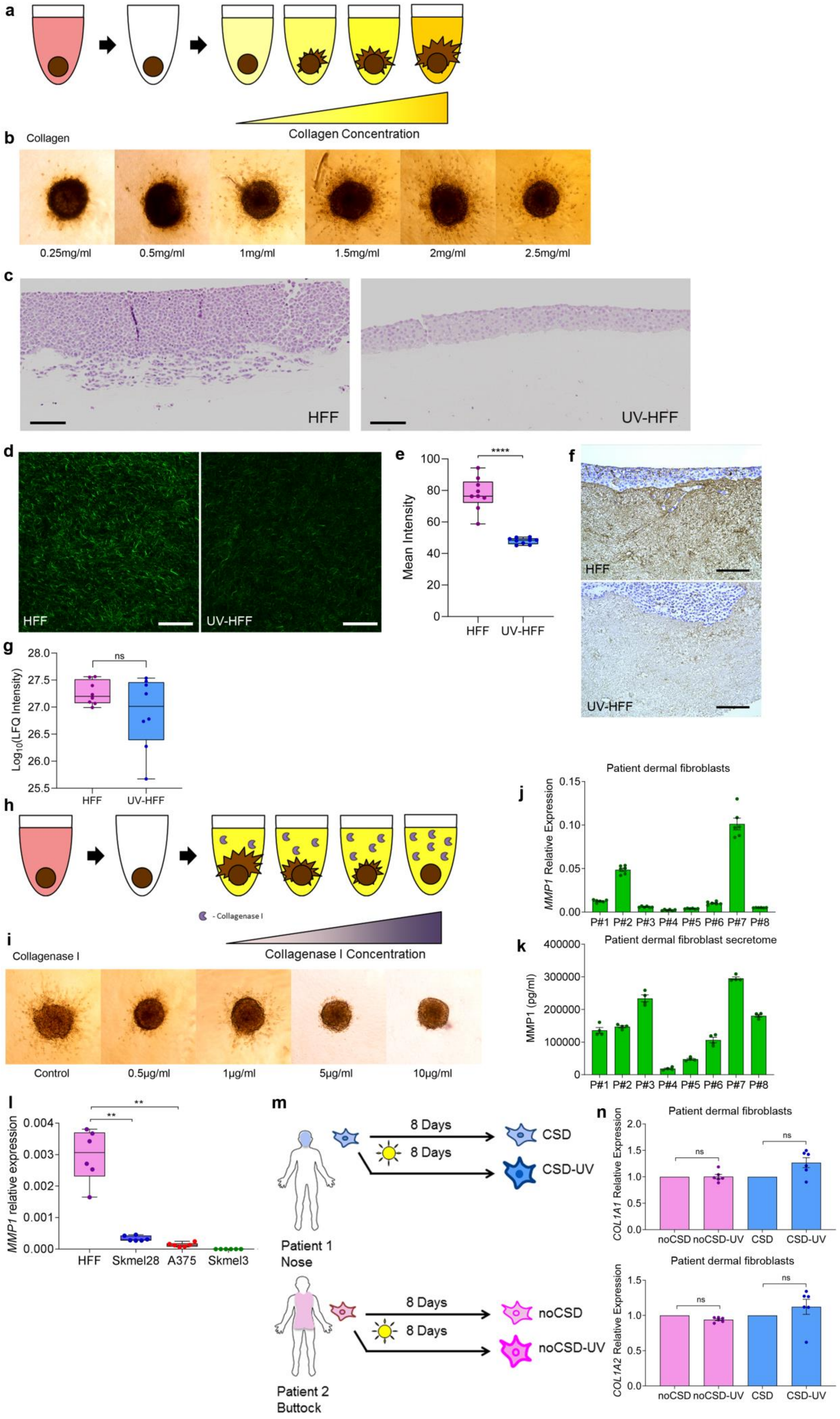
a
Collagen Concentration
b
Collagen
0.25mg/ml
0.5mg/ml
1mg/ml
1.5mg/ml
2mg/ml
2.5mg/ml
c
HFF
UV-HFF
d
HFF
UV-HFF
e
****
Mean Intensity
HFF
UV-HFF
f
HFF
UV-HFF
g
ns
Log10(LFQ Intensity)
HFF
UV-HFF
h
- Collagenase I
Collagenase I Concentration
i
Collagenase I
Control
0.5μg/ml
1μg/ml
5μg/ml
10μg/ml
j
Patient dermal fibroblasts
MMP1 Relative Expression
P#1 P#2 P#3 P#4 P#5 P#6 P#7 P#8
k
Patient dermal fibroblast secretome
MMP1 (pg/ml)
P#1 P#2 P#3 P#4 P#5 P#6 P#7 P#8
l
**
**
MMP1 relative expression
HFF
Skmel28
A375
Skmel3
m
8 Days
8 Days
CSD
CSD-UV
Patient 1
Nose
8 Days
8 Days
noCSD
noCSD-UV
Patient 2
Buttock
n
Patient dermal fibroblasts
COL1A1 Relative Expression
ns
ns
noCSD noCSD-UV CSD CSD-UV
Patient dermal fibroblasts
COL1A2 Relative Expression
ns
ns
noCSD noCSD-UV CSD CSD-UV

Supplementary Figure 2. Melanoma invasion decreases in low amounts and degraded collagen a. Graphical representation of spheroid model of collagen concentration gradient. **b.** Representative Sk-mel-28 spheroid invasion in collagen gradient. **c.** Representative H&E photomicrographs of UV and UV-HFF constructs with melanoma cells,scale bar: 150 µm. **d.** Second harmonic generation (SHG) imaging of collagen fibres in organotypic dermal collagen HFF and UV-HFF constructs, scale bar: 50 µm. **e.** Quantification of collagen from SGH images in HFF and UV-HFF, (two-sided Mann Whitney U ****$p<0.0001$). **f.** Fibronectin IHC staining in organotypic dermal collagen HFF and UV-HFF constructs, scale bars: 50 µm. **g.** Label free quantification (LFQ) of elastin (ELN) in the HFF and UV-HFF matrisome by mass spectrometry (two-sided Mann Whitney U ns: not significant). **h.** Graphical representation of spheroid model of collagenase I concentration gradient. **i.** Representative Sk-mel-28 spheroid invasion in collagenase I gradient. **j.** Relative expression of *MMP1* in panel of eight healthy adult dermal fibroblasts cell lines. **k.** Quantification of MMP1 in the secretome of eight adult fibroblast cell lines. **l.** Relative expression of *MMP1* in melanoma cell lines (Sk-mel-28, A375, Sk-mel-3) compared to HFF fibroblasts, (two-sided Mann Whitney U **$p=0.0022$). **m.** Graphical representation of isogenic chronic UVR model in two adult fibroblast lines. **n.** Fold change in expression of *COL1A1* and *COL1A2* in chronic UVR fibroblasts normalised to isogenic untreated cell lines, (two-sided Mann Whitney U ns: not significant). Error bars: standard error of the mean (bar). Box plots represent 25th to 75th percentiles with median, whiskers represent minimum and maximum values.

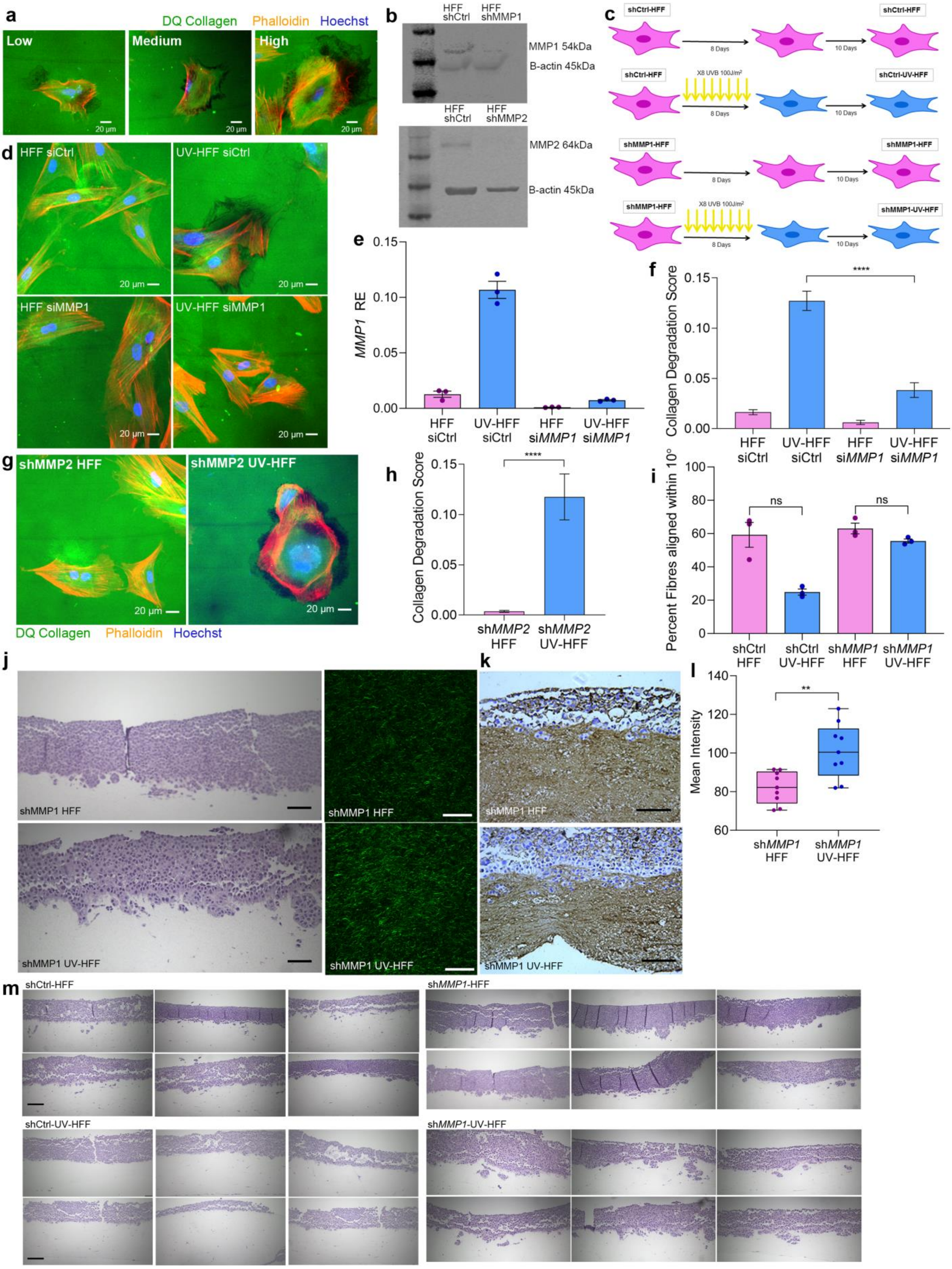
a
DQ Collagen Phalloidin Hoechst
Low
Medium
High
20 µm
b
HFF shCtrl
HFF shMMP1
MMP1 54kDa
B-actin 45kDa
HFF shCtrl
HFF shMMP2
MMP2 64kDa
B-actin 45kDa
c
shCtrl-HFF
8 Days
10 Days
X8 UVB 100J/m²
shCtrl-UV-HFF
shMMP1-HFF
shMMP1-UV-HFF
d
HFF siCtrl
UV-HFF siCtrl
HFF siMMP1
UV-HFF siMMP1
e
MMP1 RE
HFF siCtrl
UV-HFF siCtrl
HFF siMMP1
UV-HFF siMMP1
f
Collagen Degradation Score
****
g
shMMP2 HFF
shMMP2 UV-HFF
DQ Collagen Phalloidin Hoechst
h
Collagen Degradation Score
shMMP2 HFF
shMMP2 UV-HFF
i
Percent Fibres aligned within 10°
ns
shCtrl HFF
shCtrl UV-HFF
shMMP1 HFF
shMMP1 UV-HFF
j
shMMP1 HFF
shMMP1 UV-HFF
k
l
Mean Intensity
**
shMMP1 HFF
shMMP1 UV-HFF
m
shCtrl-HFF
shMMP1-HFF
shCtrl-UV-HFF
shMMP1-UV-HFF

Supplementary Figure 3. UVR damage to human dermal fibroblasts upregulate MMP1 but not COL1A1 a Representative images of collagen degradation scores as outlined in methods. **b** Western blots validating knockdown of MMP1 and MMP2 in shRNA cell lines. **c** Graphical representation of isogenic chronic UVR model in shCtrl and sh*MMP1* HFF fibroblasts. **d** Representative images of collagen degradation in siCtrl-HFF, siCtrl-UV-HFF, si*MMP1*-HFF and si*MMP1*-UV-HFF fibroblasts. Green: intact DQ collagen; red: phalloidin; blue: Hoechst. Size bars: 20 µm. **e** Validation of siRNA effect on *MMP1* relative expression (RE) by qPCR in collagen degradation assay. **f** Quantification of collagen degradation of siCtrl-HFF and si*MMP1*-HFF (pink) and their isogenic chronic UVR cell lines siCtrl-UV-HFF and si*MMP1*-UV-HFF (blue), (two-sided Mann Whitney U ****p<0.0001). **g** Representative images of collagen degradation in sh*MMP2*-HFF, and sh*MMP2*-UV-HFF fibroblasts. Green: intact DQ collagen; red: phalloidin; blue: Hoechst. Scale bars: 20 µm. **h** Quantification of collagen degradation of sh*MMP2*-HFF and their isogenic chronic UVR cell line sh*MMP2*-UV-HFF (blue), (two-sided Mann Whitney U ****p<0.0001). **i** Quantification of fibres within 10° of mode orientation in shCtrl-HFF, shCtrl-UV-HFF, sh*MMP1*-HFF and sh*MMP1*-UV-HFF derived ECM by fibronectin immunofluorescence (shCtrl-HFF vs shCtrl-UV-HFF p=0.1 (n=3), ns: not significant) **j** Representative H&E images of sh*MMP1*-HFF and sh*MMP1*-UV-HFF fibroblasts, scale bars: 75 µm (left) and second harmonic generation (SHG) imaging of collagen fibres in organotypic dermal collagen sh*MMP1*-HFF and sh*MMP1*-UV-HFF constructs (right), scale bars: 50 µm. **k** Fibronectin IHC staining in organotypic dermal collagen sh*MMP1*-HFF and sh*MMP1*-UV-HFF constructs, scale bars: 50 µm. **l** Quantification of collagen from SGH images in sh*MMP1*-HFF and sh*MMP1*-UV-HFF (two-sided Mann Whitney U, **p=0.004). **m** Representatives H&E photomicrographs of shCtrl-HFF (four top left images), shCtrl-UV-HFF (four top right images), sh*MMP1*-HFF (four bottom left images) and shMMP1-UV-HFF (four bottom right images) derived ECM constructs with invading melanoma cells, scale bars: 100 µm, scale for all images equal. Error bars: standard error of the mean (bar). Box plots represent 25th to 75th percentiles with median, whiskers represent minimum and maximum values.

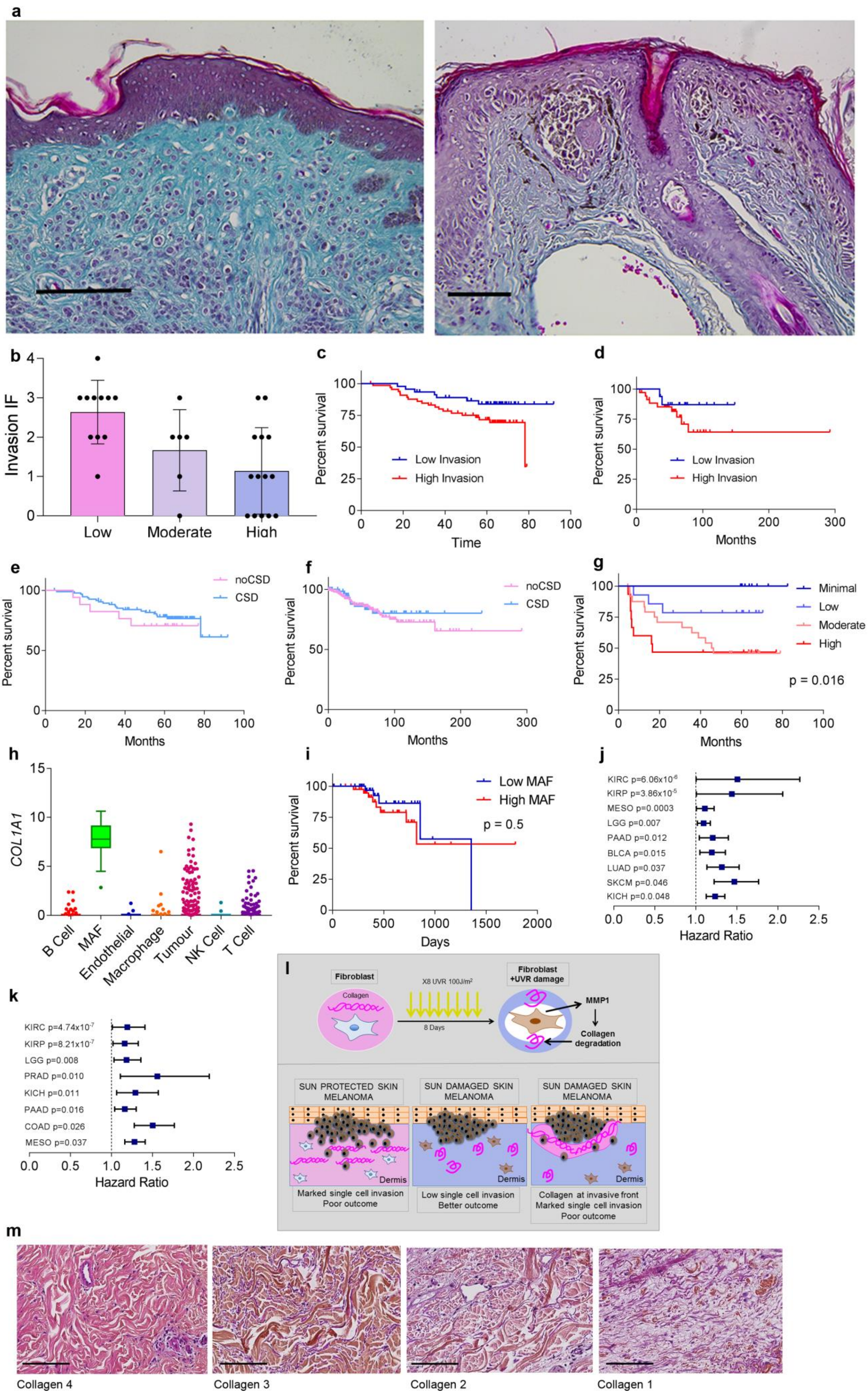
a
b
Invasion IF
4
3
2
1
0
Low
Moderate
High
c
Percent survival
100
75
50
25
0
Low Invasion
High Invasion
0
20
40
60
80
100
Time
d
Percent survival
Low Invasion
High Invasion
0
100
200
300
Months
e
Percent survival
noCSD
CSD
100
50
0
Months
f
Percent survival
noCSD
CSD
Months
g
Percent survival
Minimal
Low
Moderate
High
p = 0.016
Months
h
COL1A1
15
10
5
0
B Cell
MAF
Endothelial
Macrophage
Tumour
NK Cell
T Cell
i
Percent survival
Low MAF
High MAF
p = 0.5
0
500
1000
1500
2000
Days
j
KIRC p=6.06x10-6
KIRP p=3.86x10-5
MESO p=0.0003
LGG p=0.007
PAAD p=0.012
BLCA p=0.015
LUAD p=0.037
SKCM p=0.046
KICH p=0.0.048
0.0
0.5
1.0
1.5
2.0
2.5
Hazard Ratio
k
KIRC p=4.74x10-7
KIRP p=8.21x10-7
LGG p=0.008
PRAD p=0.010
KICH p=0.011
PAAD p=0.016
COAD p=0.026
MESO p=0.037
0.0
0.5
1.0
1.5
2.0
2.5
Hazard Ratio
l
Fibroblast
Collagen
X8 UVR 100J/m2
8 Days
Fibroblast +UVR damage
MMP1
Collagen degradation
SUN PROTECTED SKIN MELANOMA
SUN DAMAGED SKIN MELANOMA
SUN DAMAGED SKIN MELANOMA
Dermis
Dermis
Dermis
Marked single cell invasion Poor outcome
Low single cell invasion Better outcome
Collagen at invasive front Marked single cell invasion Poor outcome
m
Collagen 4
Collagen 3
Collagen 2
Collagen 1

Supplementary Figure 4. Collagen at the invasive front or primary invasive cutaneous melanoma drives melanoma invasion and poor outcome a Trichrome of Masson stain (blue) of collagen in sun protected skin (left, scale bar 200 µm) and sun damaged skin (right, scale bar 200 µm). **b** Correlation between ECM degradation of the adjacent dermis and invasive score of melanoma tumour cells at the invasive front (IF) in the A cohort. **c** Kaplan-Meier of melanoma specific survival (MSS) in prominent (high, red) and minimal (low, blue) melanoma invasion at the IF (C cohort, n=112). **d** Kaplan-Meier of melanoma specific survival (MSS) in prominent (high, red) and minimal (low, blue) melanoma invasion at the IF (B cohort, n=51). **e** Kaplan-Meier of MSS in melanoma invading in chronic sun damaged (CSD, blue) and no chronic sun damage (noCSD, pink) dermis (C cohort, n=113). **f** Kaplan-Meier of MSS in melanoma invading in CSD (blue) and noCSD (pink) dermis (B cohort, n=216). **g** Kaplan-Meier of progression free survival by collagen quantity at the IF (two-sided Log Rank test, C cohort, n=63). **h** *COL1A1* expression by cell type in metastatic melanoma single cell RNA-seq (MAF: melanoma associated fibroblast, NK cell: natural killer cell, Tirosh *et al.* Science 2016). **i** Kaplan-Meier of MSS by melanoma associated fibroblast (MAF) signature score without collagen genes in aged (>54) primary cutaneous melanoma cohort (two-sided Log Rank test, TCGA cohort, n=80). **j** Hazard ratio (centre) and 95% CI (bars) for OS, and PFS **k** Univariate Cox regression of *COL1A1* expression by cancer type in PANCAN TCGA in aged population (≥55 years) (BLCA, Bladder urothelial carcinoma, COAD, Colon adenocarcinoma, KICH, Kidney chromophobe, KIRC, Kidney renal clear cell carcinoma, KIRP, Kidney renal papillary cell carcinoma, LGG, Brain lower grade glioma, LUAD, Lung adenocarcinoma, MESO, Mesothelioma, PAAD, Pancreatic adenocarcinoma PRAD, Prostate adenocarcinoma, SKCM, Skin Cutaneous Melanoma). **l** Graphic summary of study. Risk tables for all Kaplan-Meier analyses in Supplementary Data 2. Error bars: standard error of the mean (bar). **m** Representative photomicrographs of collagen scoring. 4: preservation of collagen fibres in the dermis. 3. Combination of preserved, normal collagen bundles (pink) intermixed with elastotic fibres of non-collagenous material (purple). 2. Combination of some collagen (pink) with purple elastotic fibres. Multiple fragmentation of collagen. 1. Scarce or absent collagen, substitution of the matrix by elastotic heterogeneous fibres (purple). Rare collagen fragments interspersed (pink). Scale bars: 100 µm.

Gene Set Name	# Genes in Gene Set (K)	Description	# Genes in Overlap (k)	k/K	p-value	FDR q-value
REACTOME_EXTRACELLULAR_MATRIX_ORGANIZATION	301	Extracellular matrix organization	48	0.1595	1.91E-27	2.86E-24
REACTOME_COLLAGEN_FORMATION	90	Collagen formation	25	0.2778	4.28E-21	3.21E-18
REACTOME_DEVELOPMENTAL_BIOLOGY	1104	Developmental Biology	73	0.0661	2.54E-17	1.27E-14
REACTOME_HEMOSTASIS	674	Hemostasis	54	0.0801	1.59E-16	5.96E-14
REACTOME_ASSEMBLY_OF_COLLAGEN_FIBRILS_AND_OTHER_MULTIMERIC_STRUCTURES	61	Assembly of collagen fibrils and other multimeric structures	18	0.2951	4.32E-16	1.29E-13
REACTOME_AXON_GUIDANCE	554	Axon guidance	47	0.0848	1.79E-15	4.47E-13
REACTOME_COLLAGEN_BIOSYNTHESIS_AND_MODIFYING_ENZYMES	67	Collagen biosynthesis and modifying enzymes	18	0.2687	2.73E-15	5.85E-13
REACTOME_CELL_JUNCTION_ORGANIZATION	92	Cell junction organization	20	0.2174	6.74E-15	1.26E-12
REACTOME_CELL_CELL_COMMUNICATION	130	Cell-Cell communication	22	0.1692	7.41E-14	1.23E-11
REACTOME_INTEGRIN_CELL_SURFACE_INTERACTIONS	85	Integrin cell surface interactions	18	0.2118	2.45E-13	3.68E-11

Supplementary Table 1. Top 10 Reactome pathways significantly enriched by genes differentially expressed by Signature 7 mutation count

ID	Gene	Expression Change
COL3A1	collagen type III alpha 1 chain	DOWN
COL5A1	collagen type V alpha 1 chain	DOWN
COL5A3	collagen type V alpha 3 chain	DOWN
COL4A1	collagen type IV alpha 1 chain	DOWN
COL4A2	collagen type IV alpha 2 chain	DOWN
COL1A1	collagen type I alpha 1 chain	DOWN
ITGA6	integrin subunit alpha 6	DOWN
COL10A1	collagen type X alpha 1 chain	DOWN
COL8A1	collagen type VIII alpha 1 chain	DOWN
COL8A2	collagen type VIII alpha 2 chain	DOWN
COL11A1	collagen type XI alpha 1 chain	DOWN
COL15A1	collagen type XV alpha 1 chain	DOWN
PLEC	plectin	DOWN
CD151	CD151 molecule (Raph blood group)	DOWN
MMP3	matrix metallopeptidase 3	UP
CTSL	cathepsin L	UP
LOX	lysyl oxidase	DOWN
LOXL2	lysyl oxidase like 2	DOWN
COL26A1	collagen type XXVI alpha 1 chain	DOWN
P4HB	prolyl 4-hydroxylase subunit beta	DOWN
SERPINH1	serpin family H member 1	DOWN
COLGALT2	collagen beta(1-O)galactosyltransferase 2	DOWN
PLOD1	procollagen-lysine,2-oxoglutarate 5-dioxygenase 1	DOWN
P3H2	prolyl 3-hydroxylase 2	DOWN
COLGALT1	collagen beta(1-O)galactosyltransferase 1	DOWN
ITGB1	integrin subunit beta 1	DOWN
DAG1	dystroglycan 1	DOWN
ACTN1	actinin alpha 1	DOWN
FN1	fibronectin 1	DOWN
ITGB2	integrin subunit beta 2	DOWN
MMP1	matrix metallopeptidase 1	UP
SPARC	secreted protein acidic and cysteine rich	DOWN
APP	amyloid beta precursor protein	UP
HSPG2	heparan sulfate proteoglycan 2	DOWN
COMP	cartilage oligomeric matrix protein	DOWN
ICAM2	intercellular adhesion molecule 2	DOWN
ICAM5	intercellular adhesion molecule 5	DOWN
ITGA11	integrin subunit alpha 11	DOWN
MMP8	matrix metallopeptidase 8	UP
DCN	decorin	UP
ELN	elastin	DOWN
SCUBE3	signal peptide, CUB domain and EGF like domain containing 3	DOWN
BGN	biglycan	DOWN
ASPN	asporin	DOWN
BMP2	bone morphogenetic protein 2	UP
EFEMP2	EGF containing fibulin extracellular matrix protein 2	DOWN
BMP7	bone morphogenetic protein 7	UP
MFAP5	microfibril associated protein 5	DOWN

Supplementary Table 2. Genes from Reactome Extracellular Matrix Organisation pathway differentially expressed by Signature 7 mutations

Age median (range)	69	(34-75)
Sex	6 Female	2 Male
Anatomic site	7 Limbs and trunk	1 Head and neck

Supplementary Table 3. Patient fibroblasts

Variable	Cohort A		Cohort B		Cohort C	
	(N=31)		(N=222)		(N=113)	
Age - median ± SD (range)	68.84 ± 7.87	(57-91)	69.5 ± 9.15	(55-96)	67 ± 8.86	(55 - 88)
Sex						
Male (%)	NA		140	(63.06)	63	(55.75)
Female (%)	NA		82	(36.94)	50	(44.25)
Breslow - median ± SD (range)	1.72 ±2.74	(0.1-14)	2.2 ± 2.20	(1-13.5)	2.5 ± 3.91	(1 - 35)
Site						
Trunk (%)	NA		74	(33.33)	53	(46.90)
Head&neck (%)	NA		86	(38.74)	13	(11.50)
Upper limb (%)	NA		26	(11.71)	19	(16.81)
Lower limb (%)	NA		36	(16.22)	28	(24.79)
Solar elastosis						
noCSD (%)	17	(54.8)	149	(67.12)	17	(15.04)
CSD (%)	14	(45.2)	73	(32.88)	96	(82.22)

Supplementary Table 4. Clinical cohort details

	Cox regression model											
	Univariate			Multivariate (a)			Multivariate (b)			Multivariate (c)		
Variable	HR	95% CI	P-value	HR	95% CI	P-value	HR	95% CI	P-value	HR	95% CI	P-value
Age	1.03	1.00-1.06	0.03*	1.02	0.95-1.05	0.47	1.02	0.98-1.05	0.4	1.02	0.97-1.08	0.4
Sex												
Female	ref	ref	ref	ref	ref	ref	ref	ref	ref	ref	ref	ref
Male	2.04	1.18-3.55	0.01*	1.43	0.48-3.77	0.49	2.67	1.22-5.88	0.01*	1.3	0.48-3.48	0.6
Breslow	1.06	1.03-1.10	0.0004*	1.03	0.87-1.33	0.77	1.09	1.04-1.15	0.0009*	1	0.85-1.20	0.9
CSD												
noCSD	ref	ref	ref	ref	ref	ref	-	-	-	-	-	-
CSD	0.99	0.61-1.65	0.36	0.73	0.16-1.57	0.57						
Invasion Binary												
Low	ref	ref	ref	ref	ref	ref	ref	ref	ref	-	-	-
High	2.3	1.09-4.85	0.03*	1.17	0.39-5.41	0.81	2.76	1.21-6.27	0.02*			
Invasive Front Collagen	1.9	1.15-3.15	0.01*	1.7	1.00-3.13	0.06	-	-	-	1.85	1.11-3.1	0.019*

(a) multivariate analysis with all variables
(b) multivariate analysis with clinical variables significant in univariate and invasion score
(c) multivariate analysis with clinical variables significant in univariate and collagen scores
*p<0.05

Supplementary Table 5. Cox regression of clinical and invasion variables
