# Supplementary material for: Ultraviolet light-induced collagen degradation inhibits melanoma invasion
Source: Nat Commun. 2021 May 12;12:2742. doi: 10.1038/s41467-021-22953-z (PMC8115293; doi:10.1038/s41467-021-22953-z)
Supplement: Supplementary file 3 — Description of Additional Supplementary Files [file 41467_2021_22953_MOESM3_ESM.pdf]

Description of Additional Supplementary Files

File Name: Supplementary Data 1
Description: Differentially expressed genes by COSMIC signature 7 mutation count in health adult dermal fibroblasts

File Name: Supplementary Data 2
Description: Risk tables for all Kaplan Meier analyses
