# Supplementary material for: Ultraviolet light-induced collagen degradation inhibits melanoma invasion
Source: Nat Commun. 2021 May 12;12:2742. doi: 10.1038/s41467-021-22953-z (PMC8115293; doi:10.1038/s41467-021-22953-z)
Supplement: Supplementary file 6 — Reporting Summary [file 41467_2021_22953_MOESM6_ESM.pdf]

nature research

Corresponding author(s): Amaya Viros

Last updated by author(s): 29/03/2021

Reporting Summary

Nature Research wishes to improve the reproducibility of the work that we publish. This form provides structure for consistency and transparency in reporting. For further information on Nature Research policies, see our Editorial Policies and the Editorial Policy Checklist.

Statistics

For all statistical analyses, confirm that the following items are present in the figure legend, table legend, main text, or Methods section.

n/a	Confirmed	
☐	☒	The exact sample size (*n*) for each experimental group/condition, given as a discrete number and unit of measurement
☐	☒	A statement on whether measurements were taken from distinct samples or whether the same sample was measured repeatedly
☐	☒	The statistical test(s) used AND whether they are one- or two-sided<br>*Only common tests should be described solely by name; describe more complex techniques in the Methods section.*
☐	☒	A description of all covariates tested
☐	☒	A description of any assumptions or corrections, such as tests of normality and adjustment for multiple comparisons
☐	☒	A full description of the statistical parameters including central tendency (e.g. means) or other basic estimates (e.g. regression coefficient) AND variation (e.g. standard deviation) or associated estimates of uncertainty (e.g. confidence intervals)
☐	☒	For null hypothesis testing, the test statistic (e.g. *F*, *t*, *r*) with confidence intervals, effect sizes, degrees of freedom and *P* value noted<br>*Give P values as exact values whenever suitable.*
☒	☐	For Bayesian analysis, information on the choice of priors and Markov chain Monte Carlo settings
☒	☐	For hierarchical and complex designs, identification of the appropriate level for tests and full reporting of outcomes
☐	☒	Estimates of effect sizes (e.g. Cohen's *d*, Pearson's *r*), indicating how they were calculated

Our web collection on statistics for biologists contains articles on many of the points above.

Software and code

Policy information about availability of computer code

Data collection	Data was collected using Microsoft Excel 2010 64-bit (Microsoft), atomic force microscopy collected on Bruker ScanAsyst Nanoscope 9.1, mass spectrometry data aquired with Orbitrap Fusion Lumos mass spectrometer (Thermo Fisher Scientific), collagen degradation and spheroid invasion images collected on Opera Phenix High Content Screening System (PerkinElmer, Inc.), ELISA data acquired on Spectra Max M5 plate reader (Molecular Devices), qPCR QuantStudio 3 system (Thermo Fisher), Leica Multiviews for histology, Leica SCN400 for whole slide imaging system and Aperio ImageScope software v12.3 (Leica Microsystems).
Data analysis	Mass spectrometry raw data were processed using MaxQuant software13 (version 1.6.3.3), Perseus software 16 (version 1.6.2.2) was used for statistical analysis. AFM data were processed using Nanoscope Analysis software 1.4 prior to image export. Sequencing used Trimmomatic-0.32 and STAR_2.3.0e_r291. Image Analysis for microscopy was performed using ImageJ (version 1.53c). For in vitro studies statistical analysis was performed in GraphPad Prism (version 7.01, GraphPad Software, Inc.). For clinical data studies statistical analysis was performed in R (version 3.5.1, RStudio v1.2.5001, RStudio Inc). No custom codes or algorithms were used in the analysis of this study and all analysis packages and software is cited in manuscript.

For manuscripts utilizing custom algorithms or software that are central to the research but not yet described in published literature, software must be made available to editors and reviewers. We strongly encourage code deposition in a community repository (e.g. GitHub). See the Nature Research guidelines for submitting code & software for further information.

Data

Policy information about availability of data

All manuscripts must include a data availability statement. This statement should provide the following information, where applicable:

- Accession codes, unique identifiers, or web links for publicly available datasets
- A list of figures that have associated raw data
- A description of any restrictions on data availability

RNA-seq data used in this study has been previously published and available from ENA project PRJEB13731 (https://www.ebi.ac.uk/ena/browser/view/PRJEB13731). The datasets generated during the current study are available from the corresponding authors upon reasonable request.

Field-specific reporting

Please select the one below that is the best fit for your research. If you are not sure, read the appropriate sections before making your selection.

☒ Life sciences ☐ Behavioural & social sciences ☐ Ecological, evolutionary & environmental sciences

For a reference copy of the document with all sections, see nature.com/documents/nr-reporting-summary-flat.pdf

Life sciences study design

All studies must disclose on these points even when the disclosure is negative.

Sample size	For clinical data: all primary cutaneous melanomas available were included.<br>For SNV and solar elastosis scores: all available sequenced fibroblasts were included<br>For in vitro studies sample sizes were based on numbers to observe at least a 20% variance in data at 80% power, error 0.05, using a n=5-7 or greater. Melanoma spheroid assays were performed in 3 independent cell lines with 8 replicates across 3 independent experiments. 8 patient fibroblast cell lines were used for in vitro work and validated using two independent in vitro isogenic cell lines. All possible studies were done at least with 2 biological replicates and 2 technical replicates. All duplicates, technical replicates indicated in methods.
Data exclusions	No data were excluded
Replication	All isogenic UVR HFF models were made in biological duplicate and all samples collected in duplicate. All samples were run at minimum in technical duplicate where possible. All replication experiments were successful
Randomization	None of our experiments required allocation in groups for control vs intervention.
Blinding	Investigators were blinded during the scoring of melanoma IHC slides and 3D organotypic models for invasion and collagen. Invasion in 3D models with spheroids were additionally repeated by an independent lab, scored independently, blinded for collagen concentration. 3D organotypic constructs and collagen degradation assays were scored blindly by 2 independent observers., with high interobserver agreement. In cases of disagreement, a joint consensus was reached. Spheroid invasion of patient fibroblast secretomes was done blinded for MMP1 concentration in the secretome. AFM and proteomics were done blinded for the hypothesis by two independent research groups.<br>Clinical parameters were scored blinded for outcome by 2 observers (cohorts A and C), 20% of samples of cohort B were scored independently by 2 observers. Interobserver agreement >0.65 was determined. All scores were done blinded for patient survival. Observers who scored pathological specimens had no access to clinical data at any time.

Reporting for specific materials, systems and methods

We require information from authors about some types of materials, experimental systems and methods used in many studies. Here, indicate whether each material, system or method listed is relevant to your study. If you are not sure if a list item applies to your research, read the appropriate section before selecting a response.

Materials & experimental systems

n/a	Involved in the study	
☐	☒	Antibodies
☐	☒	Eukaryotic cell lines
☒	☐	Palaeontology and archaeology
☒	☐	Animals and other organisms
☐	☒	Human research participants
☒	☐	Clinical data
☒	☐	Dual use research of concern

Methods

n/a	Involved in the study	
☒	☐	ChIP-seq
☒	☐	Flow cytometry
☒	☐	MRI-based neuroimaging

Antibodies

Antibodies used	Primary antibody to Fibronectin (1:200, F3648, Sigma Aldrich)
Validation	All antibodies used were validated for the specific application and species by the manufacturers and all antibodies were used according the manufactures instructions only for the validated species and application. Additionally, we validated staining in human skin as a positive control alongside an isotype negative control antibody.

Eukaryotic cell lines

Policy information about cell lines

Cell line source(s)	HFF purchased from ATCC (ATCC SCRC-1041). Melanoma cell lines were also purchased from ATCC Sk-mel-28 (ATCC HTB-72), Sk-mel-3 (ATCC HTB-69), and A375 (ATCC CRL-1619)
Authentication	Melanoma cell lines were authenticated by STR profiling
Mycoplasma contamination	All the cell lines tested negative for Mycoplasma in routine testing.
Commonly misidentified lines (See ICLAC register)	No commonly misidentified cell lines were used in this study

Human research participants

Policy information about studies involving human research participants

Population characteristics	Three patient cohorts of primary cutaneous melanoma from patients aged ≥55 were used in this paper, from 3 countries. The A cohort (n=31), the B cohort (n=222) and the C cohort (n=113). All clinical and pathological information assessed was done under appropriate institutional ethics approvals. Comprehensive clinical outcome was available for the B and C cohorts and was collected prospectively at both institutions. The median age of patients was A=68.84, B=69.5, C=67. The cohorts reflect the sex bias in melanoma, with a 63% and 55% male preponderance in cohorts B and C respectively. The correlation between solar elastosis and invasion of melanoma cells at the invasive front (IF) was done in the A cohort in patients with invasive primary melanoma where a distinct vertical growth was determined in patients aged ≥55 at the time of diagnosis. The correlation and histological assessments of the B and C cohorts were done in primary cutaneous melanomas of patients aged ≥55 at the time of diagnosis with Breslow ≥1mm. The clinical characteristics of the cohorts are described in Supplementary Table 4.
Recruitment	The cohorts are unselected, clinical cases prospectively collected. No patients were excluded.
Ethics oversight	A, B and C cohort: The relevant institutional review board and ethics committee, in their respective institutions.<br>Human participants gave written/oral informed consent if required by institutions. All clinical and pathological information assessed complied with all relevant ethical regulations for work with human participants in the UK, Spain and France: Salford cohort A: Local ethics and UK NHS REC regulation approval, IRAS 16/LO/2098 (16/SW/0323); no patient signed consent required; Spanish cohort B: Internal Review Board of the Instituto Valenciano Oncología in Valencia, and verbal informed consent was obtained from all patients who were alive at the time of the study; French cohort C: Internal Review Board of the Comite de Protection des Personnes Sud Méditerranée and Aix-Marseile University Hospital approval, and signed informed consent was obtained.

Note that full information on the approval of the study protocol must also be provided in the manuscript.
